# Comparative analyses reveal distinct sets of lineage-specific genes within *Arabidopsis thaliana*

**DOI:** 10.1186/1471-2148-10-41

**Published:** 2010-02-12

**Authors:** Haining Lin, Gaurav Moghe, Shu Ouyang, Amy Iezzoni, Shin-Han Shiu, Xun Gu, C Robin Buell

**Affiliations:** 1 Department of Plant Biology, Michigan State University, 166 Plant Biology Building, East Lansing, MI 48824, USA; 2 Department of Genetics, Development, and Cell Biology, Iowa State University, Ames, IA 50011, USA; 3 J. Craig Venter Institute, 9712 Medical Center Drive, Rockville, MD 20850, USA; 4 Department of Horticulture, Michigan State University, A342 Plant and Soil Science Building, East Lansing, MI 48824, USA; 5 Current address: Suite 205, 1003 7th Street, Frederick, MD 21701, USA

## Abstract

**Background:**

The availability of genome and transcriptome sequences for a number of species permits the identification and characterization of conserved as well as divergent genes such as lineage-specific genes which have no detectable sequence similarity to genes from other lineages. While genes conserved among taxa provide insight into the core processes among species, lineage-specific genes provide insights into evolutionary processes and biological functions that are likely clade or species specific.

**Results:**

Comparative analyses using the *Arabidopsis thaliana *genome and sequences from 178 other species within the Plant Kingdom enabled the identification of 24,624 *A. thaliana *genes (91.7%) that were termed Evolutionary Conserved (EC) as defined by sequence similarity to a database entry as well as two sets of lineage-specific genes within *A. thaliana*. One of the *A. thaliana *lineage-specific gene sets share sequence similarity only to sequences from species within the Brassicaceae family and are termed Conserved Brassicaceae-Specific Genes (914, 3.4%, CBSG). The other set of *A. thaliana *lineage-specific genes, the Arabidopsis Lineage-Specific Genes (1,324, 4.9%, ALSG), lack sequence similarity to any sequence outside *A. thaliana*. While many CBSGs (76.7%) and ALSGs (52.9%) are transcribed, the majority of the CBSGs (76.1%) and ALSGs (94.4%) have no annotated function. Co-expression analysis indicated significant enrichment of the CBSGs and ALSGs in multiple functional categories suggesting their involvement in a wide range of biological functions. Subcellular localization prediction revealed that the CBSGs were significantly enriched in proteins targeted to the secretory pathway (412, 45.1%). Among the 107 putatively secreted CBSGs with known functions, 67 encode a putative pollen coat protein or cysteine-rich protein with sequence similarity to the *S*-locus cysteine-rich protein that is the pollen determinant controlling allele specific pollen rejection in self-incompatible Brassicaceae species. Overall, the ALSGs and CBSGs were more highly methylated in floral tissue compared to the ECs. Single Nucleotide Polymorphism (SNP) analysis showed an elevated ratio of non-synonymous to synonymous SNPs within the ALSGs (1.99) and CBSGs (1.65) relative to the EC set (0.92), mainly caused by an elevated number of non-synonymous SNPs, indicating that they are fast-evolving at the protein sequence level.

**Conclusions:**

Our analyses suggest that while a significant fraction of the *A. thaliana *proteome is conserved within the Plant Kingdom, evolutionarily distinct sets of genes that may function in defining biological processes unique to these lineages have arisen within the Brassicaceae and *A. thaliana*.

## Background

Lineage-specific genes are defined as genes in one taxonomic group that have no detectable sequence similarity to genes from other lineages. With the availability of complete or near-complete genome and transcriptome sequences from a wide range of species, lineage-specific genes have been extensively studied, especially in microbial species [1-4]. Several hypotheses regarding the origin of lineage-specific genes have been proposed. One model suggests that lateral gene transfer has an important role in generating lineage-specific genes [5,6]. The second model proposes that lineage-specific genes may be generated by gene duplication followed by rapid sequence divergence [4,7]. It is also suggested that an accelerated evolutionary rate may be responsible for the emergence of lineage-specific genes such that no sequence similarity to genes from other species can be detected [8]. Other models include *de novo *emergence from non-genic sequences which are more diverged between species [9] as well as artifacts from genome annotation [10]. Although the origin and evolution of lineage-specific genes remains unresolved, the identification and characterization of putative lineage-specific genes can provide insight into species-specific functions and evolutionary processes such as speciation (divergence) and adaptation [4].

Within the Plant Kingdom, the identification and characterization of lineage-specific genes has been performed through comparative analysis of Expressed Sequence Tags (ESTs) and/or the finished genome sequences of *Arabidopsis thaliana *(Arabidopsis) and *Oryza sativa *(rice) [11-13], the model species for dicotyledonous and monocotyledonous plants, respectively. Through a comparative analysis between the Arabidopsis and rice predicted proteomes, 116 protein clusters comprised of at least two Arabidopsis sequences but lacking a rice protein were identified, suggesting they were encoded by Arabidopsis-specific genes [14,15]. In a comparative analysis of legume with non-legume unigene datasets, GenBank's nonredundant and EST databases, and the genome sequences of Arabidopsis and rice, approximately 6% of the legume unigene sets were identified as legume-specific [13]. In a more recent analysis, a set of 861 rice genes termed "Conserved Poaceae Specific Genes" that are evolutionarily conserved within the Poaceae family yet lack significant sequence similarity to non-Poaceae species was identified by searching the finished rice genome sequence against the genomic sequences from Arabidopsis, *Medicago*, poplar, and EST clusters from 184 plant species [16]. This set of conserved Poaceae-specific genes provides a starting point for further research experiments to better understand the unique morphology, physiological and developmental characteristics of Poaceae species. With the recent availability of additional plant genome sequences, a recent study identified 165, 638, and 109 lineage-specific genes in Arabidopsis, rice, and poplar, respectively, by searching genes with expression evidence against EST assemblies, a non-redundant protein database, and plant genome sequences [17]. In addition to lineage-specific genes, comparisons of multiple plant genomes can provide information on lineage-specific gene expansion of gene families [18].

In this study, we identified and characterized Conserved Brassicaceae-Specific Genes (CBSGs) and Arabidopsis Lineage-Specific Genes (ALSGs) using the completed and well-annotated *A. thaliana *genome, the genomes of *Medicago truncatula *(Medicago), *Populus trichocarpa *(poplar), *Vitis vinifera *(grapevine), *Carica papaya *(papaya), *Sorghum bicolor *(sorghum), *Chlamydomonas reinhardtii *(green alga), *Physcomitrella patens *(moss), and *O. sativa *(rice) [19-27], as well as EST clusters from 178 plant species. An earlier study on Arabidopsis lineage-specific genes [17] restricted their analysis to only genes with expression evidence and employed a relaxed criterion to define sequence conservation. As a consequence, only 165 lineage-specific genes were identified in Arabidopsis. In our study, we elected to limit false negatives and identify more potential lineage-specific genes in *A. thaliana *by using the entire *A. thaliana *predicted protein-coding gene complement in our analyses and by using more stringent searching criteria. Furthermore, we identified two types of lineage-specific genes, those restricted to *A. thaliana *and those restricted to the Brassicaceae. By our definition, CBSGs are *A. thaliana *genes that have significant sequence similarity only to sequences from species within the Brassicaceae family while ALSGs are *A. thaliana *genes that are unique to *A. thaliana*. As a large portion of the CBSGs and ALSGs have no known function, co-expression and subcellular localization analyses were performed to infer possible biological function. DNA methylation analysis was performed to investigate the epigenetic modification and effects. To assess evolutionary pressures within these two sets of lineage-specific genes, Single Nucleotide Polymorphisms (SNPs) within the coding regions were analyzed.

## Results

### Identification of CBSGs and ALSGs

**Figure 1:**
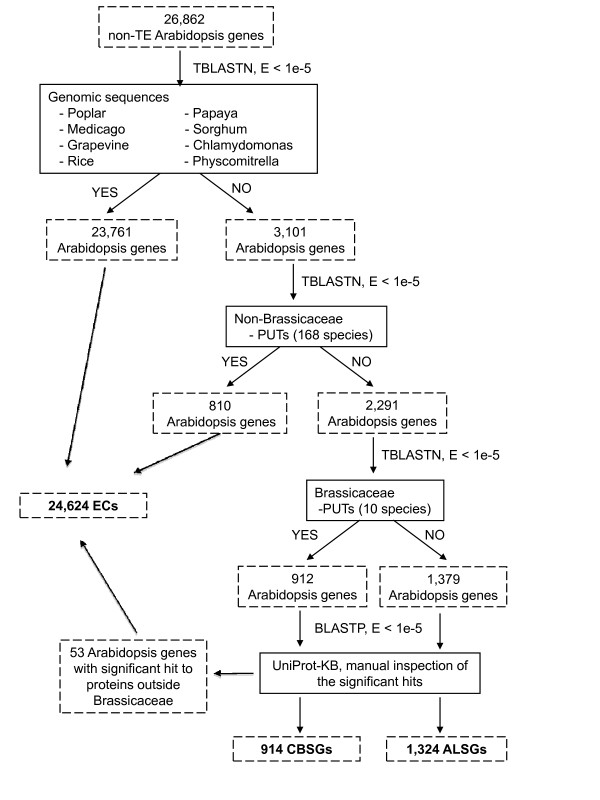
**Identification of lineage specific genes in *A. thaliana***. The solid boxes reflect non-Arabidopsis sequences used in the searches while the hashed boxes show the Arabidopsis genes.

Using TBLASTN [28], 26,862 *A. thaliana *protein-coding genes were searched against the genomic sequences of papaya, poplar, *Medicago*, grapevine, rice, sorghum, moss, *Chlamydomonas*, and the PlantGDB-assembled Unique Transcripts (PUTs) [29] from 168 species outside the Brassicaceae family. A total of 24,571 *A. thaliana *genes with significant sequence similarity (E-value < 1e-5) to either a genomic or PUT sequence from a species outside the Brassicaceae family were defined as the Evolutionarily Conserved (EC) set (Fig. 1). The remaining 2,291 *A. thaliana *genes with no significant similarity to any sequence (genomic or PUT) outside the Brassicaceae family were further searched against PUT sequences from ten Brassicaceae species/subspecies including: *Brassica napus*, *B. oleracea*, *B. oleracea *var. *alboglabra*, *B. rapa, Raphanus raphanistrum *subsp *landra, R. raphanistrum *ssp. *maritimus, R. raphanistrum *ssp. *raphanistrum, R. sativus*, *R. sativus *var. *oleiformis*, and *Thellungiella halophila*. This resulted in two datasets: 912 CBSGs with no significant sequence similarity to sequences from the Plant Kingdom except those from the Brassicaceae, and 1,379 ALSGs that had no significant sequence similarity to any sequences within the Plant Kingdom (Fig. 1). To further eliminate false positives due to incompleteness of the genome and transcriptome sequence sets, the CBSGs and ALSGs were used to search against the UniProt Knowledgebase (UniProtKB) using BLASTP [28]. Manual inspection of the alignments (E-value < 1e-5) identified 53 *A. thaliana *genes (33 CBSGs and 20 ALSGs) with sequence similarity to non-Brassicaceae UniProt entries that were transferred from the CBSG and ALSG sets to the EC set. A total of 35 ALSGs with sequence similarity to Brassicaceae UniProt entries were also removed from the ALSG set to the CBSG set. Thus, the final sets of CBSGs, ALSGs, and ECs contain 914, 1,324, and 24,624 *A. thaliana *genes, respectively (Fig. 1, Additional file 1, Additional file 2).

### Characterization of the CBSGs and ALSGs

To discern whether there are significant differences between the two lineage-specific gene sets (CBSGs, ALSGs) and the ECs and to exclude the possibility that the CBSGs and ALSGs are Transposable Element (TE)-related genes, genic features of the CBSGs and ALSGs were characterized and compared to those of the EC and TE gene sets (Table 1). The average exon numbers per gene for the CBSGs and ALSGs were similar to that of the TE set, but significantly smaller than that of the EC gene set (*t*-tests, P < 1e-5), consistent with previous findings of shorter gene size of lineage-specific genes in rice [16,30]. The average exon length of the CBSGs and ALSGs was comparable to that of the EC set, but was one fifth of that of the TE set (Table 1). The average intron length of the CBSGs and ALSGs were slightly longer than that of the EC and TE sets (*t*-tests, P < 1e-5). A total of 322 (35.2%) CBSGs, 861 (65.0%) ALSGs, and 4,719 (19.2%) ECs were single-exon genes, of which, 256 (79.5%) CBSGs, 310 (36.0%) ALSGs, and 4,388 (93.0%) ECs were expressed. The CBSGs had a lower GC content for the whole gene compared to the EC and TE genes (*t*-tests, P < 1e-5). The GC content for the whole gene of the ALSGs was higher than that of the ECs yet lower than that of the TE genes (*t*-tests, P < 1e-5). Both the CBSGs and ALSGs had a significantly lower average GC content for the coding sequence compared to the EC set (*t*-tests, P < 1e-5), with the CBSGs having the lowest GC content. The lower GC content observed for the CBSGs and ALSGs was consistent with the previous report on lower GC content of lineage-specific genes in Drosophila [4] but contrasted with the elevated GC content within the coding sequences of the Poaceae-specific genes in rice [16]. One explanation for the difference in GC content between the lineage-specific genes and EC genes in Arabidopsis versus rice is that neither the broader GC content distribution observed in rice exons nor the GC content gradient within rice coding sequences is present in *A. thaliana *[31]. Overall, genic metrics for the CBSGs and ALSGs indicate they are distinct gene sets from EC and TE-related gene sets.

**Table 1:** Genic features of the CBSGs, ALSGs, ECs, and TE-related genes

	CBSGs		ALSGs		ECs		TE-related genes	
				
Feature	Mean (SD)	Median	Mean (SD)	Median	Mean (SD)	Median	Mean (SD)	Median
Exons/gene	2.2 (1.6)	2	1.7 (1.4)	1	6.0 (5.2)	4	1.7 (2.4)	1
Exon length	256 (250)	182	213 (221)	147	280 (352)	155	1,336 (1,675)	522
Intron length	205 (263)	109	227 (321)	114	163 (172)	99	160 (186)	96
Gene length	827 (689)	598	537 (652)	261	2,315 (1,558)	1,998	2,420 (1,742)	2,072
Protein length	148 (112)	104	97 (85)	66	431 (298)	370	na	na
Exon GC (%)	41.0 (6.1)	40.7	42.3 (6.1)	42.2	42.6 (4.6)	42.6	42.7 (5.3)	42.3
Intron GC (%)	31.5 (7.4)	31.3	35.1 (7.6)	34.4	32.4 (4.4)	32.7	32.8 (7.9)	31.9
Gene GC (%)	37.8 (5.0)	37.8	41.0 (5.1)	40.9	39.6 (3.3)	39.3	41.5 (4.6)	41.4
CDS/ORF GC(%)	42.2 (4.4)	42	42.8 (4.8)	42.7	44.5 (3.2)	44.2	na	na
1st position GC (%)	45.5 (6.7)	45.6	45.7 (7.6)	45.7	50.2 (4.7)	50.2	na	na
2nd position GC (%)	40.4 (6.5)	40	40.0 (7.7)	40	40.5 (5.4)	40.1	na	na
3rd position GC (%)	40.8 (8.4)	40.9	42.8 (8.1)	42.9	42.9 (6.3)	42.1	na	na

With respect to function, both the CBSG and ALSG gene sets are enriched in genes of unknown function with 696 CBSGs (76.1%) and 1,250 ALSGs (94.4%) encoding proteins with no known function (Table 2). However, a large portion of both the CBSG and ALSG gene sets have transcript support from ESTs, cDNAs, or microarray data, providing increased confidence in their annotations (Table 2). A total of 68 CBSGs (7.4%) encode low-molecular-weight, cysteine-rich (LCR) proteins or S locus cysteine-rich like (SCRL) proteins, which have sequence similarity to members of the pollen coat protein (PCP) gene family or the S locus cysteine-rich protein (SCR), respectively [32]. SCR (also designated SP11) [33-35] is the pollen determinant of allele specific pollen rejection in self-incompatible Brassicaceae species. Interaction of SCR, which is localized primarily in the pollen coat, with its cognate stigma determinant, an *S*-locus receptor kinase (SRK) localized in the stigma epidermal cells [36], triggers a signal transduction cascade that results in inhibition of pollen hydration, germination, and tube growth [37]. At least one member of the PCP gene family in *Brassica *is also believed to interact with a secreted glycoprotein that is expressed specifically in the stigmatic papilla cells [38].

**Table 2:** Functional annotation of CBSGs, ALSGs, and ECs

	CBSGs		ALSGs		ECs	
			
	No. of genes	Percentage^b^	No. of genes	Percentage^b^	No. of genes	Percentage^b^
With no known function	696	**76.1**	1,250	**94.4**	5,090	**20.7**
transcript support	549	60.1	641	48.4	4,904	19.9
no transcript support	147	16.1	609	46.0	186	0.8
With a known function	218	**23.9**	74	**5.6**	19,534	**79.3**
transcript support	152	16.6	59	4.5	18,699	75.9
no transcript support	66	7.2	15	1.1	835	3.4
putative PCP or SCR^a^	68	7.4	4	0.3	41	0.2
beta-galactosidase	0	0.0	13	1.0	34	0.1
other	150	16.4	57	4.3	19,459	79.0

Total	914	100.0	1,324	100.0	24,624	100.0

^a^PCP (pollen coat protein) gene family or SCR (*S *locus cysteine-rich protein)

^b^Percentages in bold represent subtotals of the CBSG, ALSG, or EC set.

Neither the CBSGs nor ALSGs were distributed randomly within the *A. thaliana *genome (See Additional file 3). Large numbers of CBSGs, ALSGs, and ECs were located within segmentally duplicated blocks consistent with the substantial segmental duplication that occurred in *A. thaliana *[39]. However, the CBSGs and ALSGs were located more frequently in non-segmentally duplicated regions compared to the ECs. A total of 23.7% and 27.3% of the CBSGs and ALSGs, respectively, were located within non-segmentally duplicated regions, compared to 13.8% EC genes (χ^2 ^test, P < 1e-5). This could be due to differential gene loss of lineage-specific genes (ALSGs, CBSGs) in segmentally duplicated versus non-segmentally duplicated regions or alternatively that the ALSGs and CBSGs are located in segmentally duplicated blocks which have rapidly evolved and thus are not detected using similarity based segmental duplication methods.

We utilized a computational pipeline in which Pfam and novel BLASTP-based protein domains (see Methods) were used to classify paralogous families. As our pipeline involves identification of novel BLASTP-based domains, proteins without a Pfam domain can also be classified into paralogous families thereby removing any bias associated with lack of a characterized protein domain. At the whole genome level, 17,911 *A. thaliana *genes were classified into 3,051 paralogous families (66.7%). For the lineage-specific gene sets, 389 CBSGs (42.6%) and 65 ALSGs (4.9%) were classified into paralogous families, substantially lower than that of the EC set (70.9%). This is consistent with what was reported for lineage-specific genes within the Poaceae [16] and consistent with previous analyses in *A. thaliana *which demonstrated paralogous families were enriched in genes with known function yet the single-copy gene complement was enriched in genes with no known function [40].

### CBSGs are enriched with proteins targeted to the secretory pathway

To provide additional levels of functional annotation of the CBSGs and ALSGs, we used the TargetP [41] program to predict the subcellular localization of the predicted *A. thaliana *proteome. TargetP determines the putative subcellular localization based on the presence of chloroplast transit peptide, mitochondrial targeting peptide, or secretory pathway signal peptide. Consistent with previous reports [42], 14.9%, 11.7%, and 20.2% of the total proteome was predicted to be targeted to the chloroplast, mitochondrion, and secretory pathway, respectively. A dramatic enrichment of proteins targeted to the secretory pathway was observed in the CBSG set (45.1%), among which, only 107 (26.0%) have a putative function (Table 3). Based on the TAIR8 assigned functions of these 107 CBSGs, 84 are likely targeted to the secretory pathway including 67 proteins similar to PCP/SCR, eight defensin-like family proteins, five putative ligands, and four Rapid Alkalinization Factor (RALF)-like proteins. As proteins involved in the secretory pathway (e.g., receptor-ligand signaling proteins, transporters, and extracellular signaling proteins) play fundamental roles in various aspects of plant functions, the finding that the majority of the secreted CBSGs have no known function suggests that Brassicaceae species possess one or more biological processes that are either specific to the Brassicaceae family or have diverged significantly from species outside the Brassicaceae family. No bias was seen in the ALSG set for proteins targeted to the secretory pathway.

**Table 3:** Subcellular localization of the CBSGs, ALSGs, ECs, and TAIR8 non-TE protein-coding genes

	No. of genes (%)	No. of known genes	No. of expressed genes
**CBSGs**			
Chloroplast	37 (4.0)	7	30
Mitochondrion	70 (7.7)	11	62
Secretory pathway	412 (45.1)	107	285
Other	395 (43.2)	93	324
Uncertain	0 (0.0)	0	0
Total	914 (100.0)	218	701
**ALSGs**			
Chloroplast	61 (4.6)	4	45
Mitochondrion	229 (17.3)	10	109
Secretory pathway	271 (20.5)	11	130
Other	763 (57.6)	49	416
Uncertain	0 (0.0)	0	0
Total	1,324 (100.0)	74	700
**ECs**			
Chloroplast	3,909 (15.9)	3,023	3,837
Mitochondrion	2,834 (11.5)	2,149	2,773
Secretory pathway	4,751 (19.3)	3,838	4,498
Other	13,117 (53.5)	10,515	12,482
Uncertain	13 (0.1)	9	13
Total	24,624 (100.0)	19,534	23,603
**TAIR8 non-TE Protein-coding Genes**			
Chloroplast	4,007 (14.9)	3,034	3,912
Mitochondrion	3,133 (11.7)	2,170	2,944
Secretory pathway	5,434 (20.2)	3,956	4,913
Other	14,275 (53.1)	10,657	13,222
Uncertain	13 (0.0)	9	13
Total	26,862 (100.0)	19,826	25,004

Although mitochondria and chloroplasts have conserved functions throughout the Plant Kingdom and conserved sets of nuclear-encoded proteins across taxa have been documented (for example, [43-45]), both computationally predicted and empirically determined proteomes of mitochondria and chloroplasts have shown the presence of lineage-specific proteins [46,47]. For example, experimental analysis of the rice mitochondrial proteome suggested that approximately 20% of the rice mitochondrial proteome may be lineage-specific as no clear homolog was detected in the Arabidopsis mitochondrial proteome [46]. Consistent with these reports, we observed a significant difference in the percentage of genes that encode proteins targeted to the mitochondrion between the CBSG, ALSG, and EC sets. The CBSGs had a significantly lower than expected percentage of genes encoding proteins targeted to the mitochondrion and the ALSGs had a significantly higher than expected percentage (χ^2 ^test, P < 1e-5). With respect to targeting to the chloroplast, CBSGs and ALSGs were detected although at a significantly lower percentage compared to the EC set (Table 3, χ^2 ^test, P < 1e-5). In sharp contrast to the EC set, the majority of these putative mitochondrial and chloroplast targeted ALSGs and CBSGs have no known function (Table 3), suggesting these lineage-specific genes may encode novel functions within these two organelles.

### Functional inference by co-expression analyses

Given the lack of functional assignment for a large percentage of the ALSG and CBSG sets, we performed co-expression analyses to associate these lineage-specific genes with genes with annotated functions in Gene Ontology (GO) functional categories [48]. To prevent ascertainment bias, GO annotation solely based on expression evidence was excluded. Using Arabidopsis ATH1 microarray expression data, we computed Pearson's Correlation Coefficients (PCC) for the ALSGs and CBSGs in comparison to all other genes on the microarray. Probes for 345 (26%) ALSGs and 314 (34%) CBSGs are present on the ATH1 array. Based on a simulation study, we found that 99% of randomly selected *A. thaliana *gene pairs have a PCC < 0.6. Using a 0.6 PCC value as the cutoff, 260 ALSGs (75%) and 250 CBSGs (80%) with microarray probes were regarded as co-expressed with ≥ 1 gene(s) with GO annotations.

With the co-expression and GO annotation information, we then determined if any GO categories were over-represented among genes co-expressed with the ALSGs or CBSGs. We found that 75 out of the 260 ALSGs (29%) and 138 out of the 250 CBSGs (55%) had ≥ 1 significantly enriched GO categories (See Methods and Additional files 4 and 5). Upon closer inspection, 35 out of 138 (25%) CBSGs were associated with GO categories related to pollen, meiosis and sexual reproduction while 34 CBSGs (25%) were associated with GO categories related to intra-cellular transport and secretory pathways (See Additional files 6, 7 and 8). Analysis using the Fisher Exact Test revealed a highly significant over-representation of genes in these categories in CBSG versus the non-CBSG datasets. These observations, despite being obtained from a subset of the lineage-specific genes, are consistent with the TargetP prediction of an enrichment of CBSGs targeted to the secretory pathway.

### CBSGs and ALSGs have a higher density of cytosine methylation

**Figure 2:**
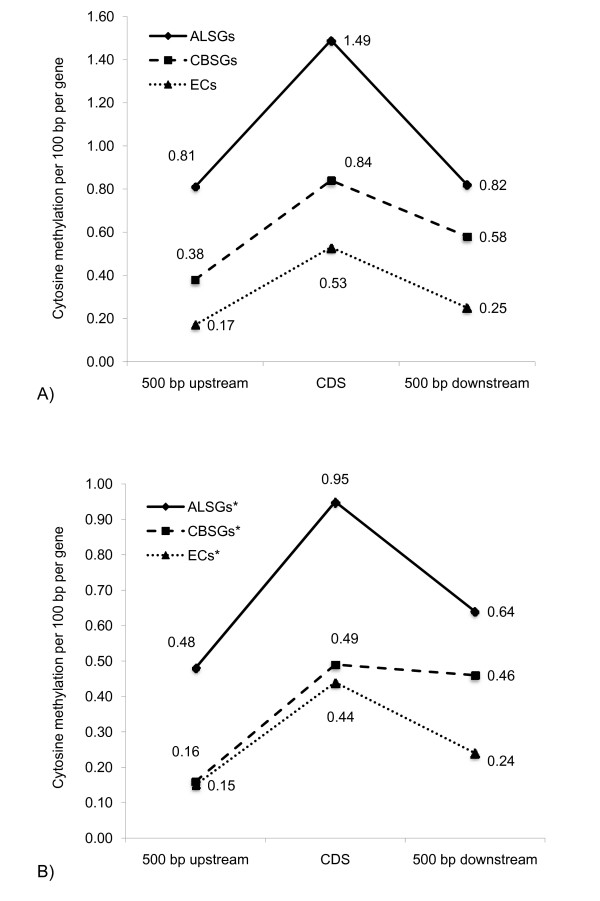
**Density of cytosine methylation in the 500 bp upstream, coding, and 500 bp downstream regions**. A) ALSGs, CBSGs, and ECs, and B) ALSGs, CBSGs, and ECs predicted to be targeted to the secretory pathway.

DNA methylation is considered one of the most important epigenetic modifications in eukaryotes. To better understand the regulation of expression of the CBSGs and ALSGs, methyl-cytosine data from floral tissue [49] was used to measure the degree of DNA methylation. More than half of DNA cytosine methylation occurs in a CG context while the remainder occurs in a CHG or CHH context (where H = A, C, T) [49]. We calculated the density of cytosine methylation for three regions of each *A. thaliana *gene: 500 bp upstream, the coding region, and 500 bp downstream. In general, the average density of methylation in the coding regions is significantly higher than that in the 500 bp upstream or downstream regions (*t*-tests, P < 1e-3). We found that the ALSGs and CBSGs had significantly more cytosine methylation than the ECs in all three regions: 500 bp upstream, coding, and 500 bp downstream regions (Fig. 2A, *t*-tests, P < 1e-5). DNA methylation in the promoter region and/or coding region of genes can repress gene expression [50], which is consistent with the fact that 95.9% of ECs compared to 52.9% of ALSGs and 76.7% of CBSGs have transcript support (Table 2). Analysis of the proteins predicted to be targeted to the secretory pathway showed decreased methylation density in all three regions compared to the full ALSG, CBSG, and EC sets (Fig. 2B, *t*-tests, P < 1e-5). For the CBSGs targeted to the secretory pathway, significantly less methylation was detected in the 500 bp upstream region compared to the full CBSG set (*t*-test, P < 1e-5), with the average methylation density (0.16 per 100 bp per gene) similar to that of the ECs (0.15 per 100 bp per gene). This suggests less suppression of expression of the secretory pathway targeted CBSGs in floral tissue.

### CBSGs and ALSGs have a higher ratio of non-synonymous to synonymous SNPs

**Figure 3:**
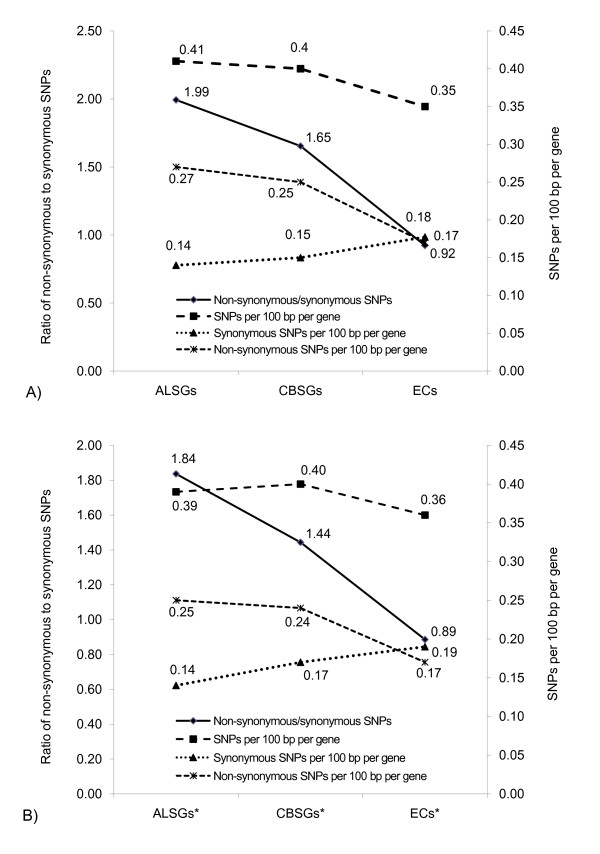
**Ratio of non-synonymous to synonymous SNPs substitutions and the number of SNPs, non-synonymous SNPs, and synonymous SNPs per 100 bp per gene within coding regions of A) ALSGs, CBSGs, and ECs, and B) ALSGs, CBSGs, and ECs predicted to be targeted to the secretory pathway**. Ratio of non-synonymous to synonymous substitutions SNPs is plotted in the solid line. Number of SNPs, non-synonymous SNPs, and synonymous SNPs per 100 bp per gene within coding regions is plotted in the dotted line. Lines are drawn between the 3 classses of genes to facilitate interpretation.

A total of 249,344 non-redundant SNPs were used to assess the genetic variation of the CBSGs and ALSGs among 20 *A. thaliana *ecotypes [51]. SNPs (243,963, 97.8%) with only a single variant base, i.e., biallelic, were used to calculate the SNP frequency within the coding regions as well as the frequencies of synonymous and non-synonymous SNPs. Taking the length of the coding sequence into account, both the ALSGs (0.41) and CBSGs (0.40) had significantly more SNPs per 100 bp per gene than the EC genes (0.35, *t*-tests, P < 1e-2). We further investigated two types of SNPs: synonymous and non-synonymous SNPs. Synonymous SNPs result in the same amino acid as the gene model from the reference genome while non-synonymous SNPs result in a different amino acid from the reference gene model. The number of non-synonymous SNPs per 100 bp per gene is significantly higher in the ALSGs (0.27) and CBSGs (0.25) compared to the ECs (0.17, *t*-tests, P < 1e-5) while the number of synonymous SNPs per 100 bp per gene is similar among the ALSG (0.14), CBSG (0.15), and EC (0.18) sets. A total of 414 (31.3%), 326 (35.7%), and 8,670 (35.3%) genes from the ALSG, CBSG, and EC sets, respectively, had more non-synonymous SNPs than synonymous SNPs. The ALSGs (1.99) and CBSGs (1.65) had greatly elevated ratios of non-synonymous to synonymous SNPs, compared to the EC set (0.92, Fig. 3A). The elevated ratios of non-synonymous to synonymous SNPs in the ALSG and CBSG sets are mainly due to the elevated non-synonymous SNP density rather than the synonymous SNP density observed in the ALSG and CBSG sets (Fig. 3A), indicating that a number of the ALSGs and CBSGs evolve substantially faster than the ECs at the protein sequence level. With respect to genes encoding proteins targeted to the secretory pathway, SNP density was comparable to that observed in the full ALSG, CBSG, and EC sets (Fig. 3B), consistent with our hypothesis that some of these genes may be involved in biological functions such as self-recognition which experience diversifying selection.

## Discussion

The 914 CBSGs and 1,324 ALSGs identified in this study are attractive targets for experimental discovery as they are lineage-specific and the majority (76.1% CBSGs and 94.4% ALSGs) encode functions yet to be determined. Both the CBSGs and ALSGs had shorter genes compared to the ECs, primarily due to fewer numbers of exons per gene and a higher percentage of single-exon genes. A total of 68.6% of the 26,862 *A. thaliana *genes used in our analyses are high confidence genes as the gene structure (including splice junctions) of at least one or more isoforms has been confirmed with a single cDNA or multiple overlapping cDNAs [27,52]. The percentages of high confidence genes within the ALSG, CBSG, and EC sets are 18.5%, 38.1%, and 72.4%, respectively. However, 54.0% and 83.9% of ALSGs and CBSGs, respectively, have transcript evidence from full length-cDNA, ESTs or microarray data, or have a putative function assigned, which provides strong support that they are likely to be bona fide genes rather than false positive gene predictions from the *ab initio *gene prediction programs utilized in genome annotation processes.

One question regarding these lineage-specific genes is their origin(s). One possibility is horizontal gene transfer. In the final step of our pipeline (Fig. 1), we utilized UniProtKB to filter any remaining conserved genes and identified 53 *A. thaliana *proteins with sequence similarity to genes from bacteria, viruses, nematodes, fungi, animals, or other plant species not available in our other large-scale plant genome and transcriptome sequence datasets. This limited number, which includes matches to other plant species, suggests that lateral gene transfer is a not a major source of lineage-specific genes in *A. thaliana*. Another potential source of lineage-specific genes is gene duplication followed by rapid evolution. Consistent with this, we found that a large number of ALSGs (73%) and CBSGs (76%) were located within segmentally duplicated regions suggesting they may have been generated by segmental duplication followed by rapid sequence divergence due to relaxed selective pressure on duplicated genes.

We have identified more lineage-specific genes (1,324 ALSGs) than the 165 Arabidopsis species-specific genes (ASS) identified by Yang *et al*. [17]. Comparison of the ASS with our ALSGs and CBSGs revealed that 10 of the ASS were transposable element genes or miRNAs and thus were not included in our analysis. Of the remaining 155 ASS described in Yang *et al*., 129 were identified as either a ALSG or a CBSG. There are two major reasons for this difference. First, different BLAST E value cutoff were utilized in the lineage-specific gene identification pipelines. Our pipeline used a BLAST E-value cutoff of < 1e-5 while Yang *et al*. [17] used a BLAST E-value cutoff of < 0.1. Because the cutoff in earlier study is relaxed, it likely has a higher false negative rate in identifying truly lineage-specific genes compared to this study while our lineage-specific gene sets will likely have higher proportion of false positives. Secondly, Yang *et al*. [17] restricted their analysis to genes with expression evidence from ESTs or full length-cDNAs. As a consequence, lineage-specific genes that were expressed under limited conditions or whose expression level was too low to be detected by traditional transcript profiling were excluded. This practice also leads to false negatives because there is ample evidence that genes annotated as hypothetical are bona-fide genes [53]. In contrast, we examined the entire *A. thaliana *predicted protein-coding gene complement and our approach likely has a significantly lower number of false negatives.

Both the ALSGs and CBSGs have more genetic variation among the 20 re-sequenced *A. thaliana *ecotypes than the EC genes, with the ALSGs and ECs having the most (0.41) and least (0.35) SNPs per 100 bp per gene, respectively. This was inversely correlated with the degree of evolutionary conservation of the ALSG, CBSG, and EC sets within the Plant Kingdom. However, the three sets have similar synonymous SNP density. As a consequence, ALSGs and CBSGs have higher ratios of non-synonymous to synonymous SNPs compared to the EC genes, indicating they are fast-evolving at the protein level.

The dramatic enrichment of secretory proteins in the CBSGs indicates there may be specific or highly evolved secretion processes within the Brassicaceae family as no significant sequence similarity could be detected in other dicot genomes including poplar, *Medicago*, papaya, and grapevine for which genome sequences are available. A majority of Brassicaceae species share a highly specialized self-incompatibility system that restricts self-pollination and promotes out-crossing [54,55] through the ability to recognize and reject self-pollen or pollen from closely related plants. In our analysis, the majority of the Brassicaceae species used are self-incompatible (*B. rapa*, *B*. *oleracea*, *B. oleracea *var. *alboglabra*, *R. raphanistrum*, and *R. sativus*) while three are self-compatible (*T. halophila*, *B. napus*, and *A. thaliana*). Within each self-incompatible *Brassica *species, specificity of the self-incompatibility response is genetically determined by the alleles at the *S *(self-incompatibility) locus and involves the arrest of pollen development upon self pollination [56]. SCR is the male determinant of the self-incompatibility response which is expressed specifically in the anther tapetum and microspores [33] and is predicted to interact with the female determinant *S *locus receptor kinase gene expressed in the papillar cells of the stigma [36]. Out-crossing is thought to be the ancestral mode of mating in the Brassicaceae [57] and the *S*-locus specificity genes are predicted to be derived from common ancestors [58].

In addition to the self-recognition mechanisms required to avoid inbreeding within a single species, plants have mechanisms to control mating between different species [59]. For example, *B. napus *pollen placed on *B. oleraceae *stigmas became hydrated and germinated; however, the pollen produced short coiled tubes that failed to penetrate the papillar cell wall [60]. This phenotype suggests that discrimination between desirable and undesirable pollen between species also involves biochemical interactions on the stigma. Therefore, it is possible that some of the CBSG PCP and SCR-like members may play a role in inter-specific mating by influencing critical aspects required for successful pollination. Our finding that 67 of the 107 CBSGs with an assigned function and putatively involved in secretory pathways are similar to SCR or PCP proteins, and that these genes are subjected to diversifying selection, a phenomena almost always associated with genes involved in recognition events, supports this hypothesis. For example, SLR1 (for *S *locus glycoprotein-like receptor 1), a stigma-specific protein, interacts with members of the PCP [38]. Whether any of the other members of the PCP or SCR like genes contribute to pollination biology in the Brassicaceae remains to be determined.

## Conclusions

In summary, we have identified two sets of *A. thaliana *lineage-specific genes, CBSGs and ALSGs, which are specific to the Brassicaceae family and *A. thaliana*, respectively. The CBSGs are especially enriched in proteins with binding function such as receptor binding that may play a role in the self-incompatibility response. The exact functions of a majority of these lineage-specific genes remain an enigma at this time. Further biological experiments will be necessary to fully understand their functions in *A. thaliana *and Brassicaceae species.

## Methods

### Data sources and preparation

The proteome of *A. thaliana *was obtained from the TAIR8 release ftp://ftp.arabidopsis.org/home/tair/Genes/TAIR8_genome_release[27]. Pseudogenes and TE genes were excluded from the original gene set based on the TAIR8 annotation, which resulted in 27,025 protein coding genes. Further screening against two in-house transposon databases identified an additional 163 putative TE-related genes, resulting in 26,862 *A. thaliana *genes for further analysis. The repeat-masked assembled scaffolds (v1.0) of poplar (*P. trichocarpa*) were downloaded from DOE Joint Genome Institute http://genome.jgi-psf.org/Poptr1_1/Poptr1_1.download.ftp.html[23]. The repeat-masked assembly of the grapevine (*V. vinifera*) genome was downloaded from Genoscope http://www.genoscope.cns.fr/spip/Vitis-vinifera-whole-genome.html[24]. The release 2.0 assembly of the Medicago (*M. truncatula*) genome was downloaded from the Medicago Genome Sequence Consortium http://www.medicago.org/genome/downloads.php[25]. Release 6 pseudomolecules of rice (*O. sativa *ssp. japonica) were downloaded from the Rice Genome Annotation Project http://rice.plantbiology.msu.edu/[26]. The repeat-masked Sbi1 assembly of the sorghum (*S. bicolor*) genome was downloaded from DOE Joint Genome Institute ftp://ftp.jgi-psf.org/pub/JGI_data/phytozome/v4.0/Sbicolor/assembly/Sbi1/[21]. The repeat-masked assembly (v4.0) of *C. reinhardtii *was downloaded from DOE Joint Genome Institute http://genome.jgi-psf.org/Chlre4/Chlre4.download.ftp.html[20]. The masked assembly (v1.1) of the moss (*P. patens *ssp. patens) genome was downloaded from DOE Joint Genome Institute http://genome.jgi-psf.org/Phypa1_1/Phypa1_1.download.ftp.html[19]. The papaya (*C. papaya*) genome was downloaded from NCBI http://www.ncbi.nlm.nih.gov/sites/entrez?db=Nucleotide&cmd=Search&term=DS981520:DS984726 [PACC][22]. The PUTs from 178 plant species (excluding *A. thaliana *in this analysis) were downloaded from PlantGDB on August 11, 2009 http://www.plantgdb.org/download/download.php?dir=/Sequence/ESTcontig. UniProtKB (Release 14.6) was downloaded from UniProt ftp://ftp.ebi.ac.uk/pub/databases/uniprot/knowledgebase/.

### Genic features

The TE set was comprised of 3,900 TE genes from the TAIR8 release and 163 putative TE genes identified by screening against two in-house transposon databases. For the TE set, only three sequence files were created: gene, exon, and intron as they lack CDS or protein sequences. For each of the CBSG, ALSG, and EC set, the sequences of gene, exon, CDS, intron, and protein were either downloaded directly from the TAIR8 release or extracted from the chromosome sequences according to the coordinates provided in the GFF3 file. Perl scripts were used to calculate the exon number, length of gene, CDS, exon, intron, and protein, GC content of CDS, gene, and three codon positions.

### Construction of paralogous protein families

A total of 26,862 non-TE *A. thaliana *proteins from the TAIR8 release were used to construct paralogous protein families in the *A. thaliana *proteome using a computational pipeline that utilized Pfam [61] and novel BLASTP-based novel domains described previously [40]. In brief, Pfam domains were identified using HMMER2 [62] with scores above the trusted cutoff value. Peptide regions that were not covered by Pfam domains were clustered based on homology (>45% identity over 75 amino acids, E-value < 1e-3) derived from an all versus all BLASTP search (WU-BLASTP 2.0 MP-WashU [22-Mar-2006]) [28]. Clustered peptides were then aligned using CLUSTALW [63,64] to develop BLASTP-based domains. Paralogous protein families were then classified based on the domain composition of each protein.

### Identification of segmental duplication

A total of 26,862 non-TE *A. thaliana *proteins from the TAIR8 release were used to identify segmental duplication in the *A. thaliana *genome using a method described previously [65]. In brief, similar protein pairs were identified by all versus all BLASTP search (WU-BLASTP 2.0 MP-WashU [22-Mar-2006], parameters "V = 5 B = 5 E = 1e-10") [28], which were then used to defined segmental duplication using DAGChainer [66] with parameters "-s -I -D 100000".

### Co-expression Analyses

The ATH1 microarray compendium of 3,037 experiments (hereafter called "supercluster") was downloaded from the NASCArrays website http://affymetrix.arabidopsis.info/narrays/help/usefulfiles.html. Only the genes having probes on the ATH1 array, 345 of the 1,324 ALSGs and 314 of the 914 CBSGs, were used for further analysis. Pairwise Pearson's Correlation Coefficient was computed between all lineage-specific genes (ALSGs and CBSGs) with array data and all genes in the supercluster. The threshold value (r = 0.6) was defined as the 99 percentile of all pairwise correlation coefficients obtained during the above computation. Using this threshold, we obtained a set of co-expressed genes for each ALSG and CBSG gene tested. 260 of the 345 ALSGs and 250 of the 314 CBSGs had > = 1 unique gene with a significantly correlated expression profile. To define the potential functions of the lineage specific genes, GO annotation of *A. thaliana *genes co-expressed with ALSGs or CBSGs were used. The *A. thaliana *GO annotation was downloaded from the TAIR website [48] excluding annotations with the evidence codes IEP, IEA and RCA. For each ALSG/CBSG, we identified the enriched GO categories among the genes significantly co-expressed. The enrichment analysis is based on a Fisher Exact Test at a False Discovery Rate of 5% as defined by the Q-value program [67]. After associating GO categories with each gene, we investigated whether there is an enrichment of genes in categories related to 1) Pollen, meiosis and sexual reproduction, 2) Intra-cellular transport and secretion, 3) Photosynthesis, 4) Defense responses, and 5) Development, cell cycle and differentiation. We manually assigned the GO categories to these five groups and determined whether these groups were over-represented among the lineage specific genes versus the non-lineage specific genes using a False Discovery Rate of 1% (See Additional files 6, 7 and 8).

### Determination of subcellular localization

The subcellular localization of 32,419 protein sequences from the 26,862 *A. thaliana *protein-coding genes was identified by TargetP program [41] using plant networks and default parameters. Subcellular localization prediction with the best (lowest) Reliability Class was used to represent the subcellular localization of the deduced protein if multiple different locations were predicted for isoforms predicted for the gene. If none of the isoforms had a prediction of 'Chloroplast', 'Mitochondrion', or 'Secretory pathway', then the subcellular localization of the gene was assigned 'Other'. If multiple subcellular localizations with equal Reliability Class were predicted for the isoforms of a gene, the subcellular localization of that gene was assigned 'Uncertain'.

### Analyses of DNA methylation

The cytosine methylomic sequence data from floral tissues of wild-type *A. thaliana *were generated by sequencing-by-synthesis technology and mapped to the *A. thaliana *genome as reported previously [49]. The methylation data for the coding region, 500 bp upstream, and 500 bp downstream of all the 26,862 *A. thaliana *protein-coding genes were kindly provided by the Ecker group. The density of methylation of cytosines was defined as the number of 5-methylcytosines per 100 bp per gene. Only representative gene models were used in our analyses.

### SNP analyses

The SNP data from re-sequencing of 20 diverse *A. thaliana *accessions using high-density oligonucleotide arrays [51] was downloaded from the TAIR8 release ftp://ftp.arabidopsis.org/Polymorphisms/Perlegen_Array_Resequencing_Data_2007/SNP_predictions/. The polymorphism GFF3 file that includes the mapping information of the SNP markers was also downloaded from the TAIR8 release ftp://ftp.arabidopsis.org/Polymorphisms/TAIR8_Variation_GFF/TAIR8_GFF3_polymorphisms.gff. PERL scripts were used to parse the data and calculate synonymous and non-synonymous SNPs within protein coding regions. A total of 249,344 SNPs were downloaded. Only base calls from the MBML2 dataset [51] were used in our analyses. Base calls of 'N' were ignored. A total of 5,381 SNPs with more than two variations within all 20 accessions were excluded from our analyses. Representative models were used whenever alternative-splicing isoforms existed. SNPs that produce same amino acid as the reference codon (Columbia-0 ecotype) was counted as synonymous SNPs while SNPs that produce a different amino acid than the reference codon was counted as non-synonymous SNPs.

## Authors' contributions

HL designed the study, conducted the majority of the computational analyses, and drafted the paper. GM and SS carried out the co-expression analysis. SO generated the additional data file 3. AI assisted in the analyses. XG supervised the analyses of SNPs and the study. CRB designed the study, supervised the study, and drafted the paper. All authors read and approved the final manuscript.

## Supplementary Material

Additional file 1 **List of CBSG genes**. The gene accession, number of exons (calculated from the representative gene model), and putative function from the TAIR8 release of all the CBSG genes are provided. Click here for file

Additional file 2 **List of ALSG genes**. The gene accession, number of exons (calculated from the representative gene model), and putative function from the TAIR8 release of all the ALSG genes are provided. Click here for file

Additional file 3 **Distribution of the CBSGs, ALSGs, and ECs within the *A. thaliana *genome**. The five *A. thaliana *chromosomes are shown with the CBSGs, ALSGs, and ECs plotted in purple, red, and blue from top to bottom, respectively. Segmentally duplicated blocks are indicated in green and the estimated centromeric regions are denoted by a yellow box. Click here for file

Additional file 4 **GO category assignments for 75 ALSGs**. Gene name, GO-ID, GO-Term, Fisher Exact Test table, p-values and Q-values for each of the 75 ALSGs with their over-represented GO categories. Click here for file

Additional file 5 **GO category assignments for 138 CBSGs**. Gene name, GO-ID, GO-Term, Fisher Exact Test table, p-values and Q-values for each of the 138 CBSGs with their over-represented GO categories. Click here for file

Additional file 6 **Grouping of GO categories into five groups**. The five defined GO category groups and their content GO IDs and GO terms. Click here for file

Additional file 7 **Results of Fisher Exact Test of ALSGs classified into groups**. Group name, Fisher Exact Test table, p-values and Q-values for each of the five GO category-groups for ALSGs. Click here for file

Additional file 8 **Results of Fisher Exact Test of CBSGs classified into groups**. Group name, Fisher Exact Test table, p-values and Q-values for each of the five GO category-groups for CBSGs. Click here for file
