# Supplementary figures and images for: Comparative analyses reveal distinct sets of lineage-specific genes within Arabidopsis thaliana
Source: BMC Evol Biol. 2010 Feb 12;10:41. doi: 10.1186/1471-2148-10-41 (PMC2829037; doi:10.1186/1471-2148-10-41)

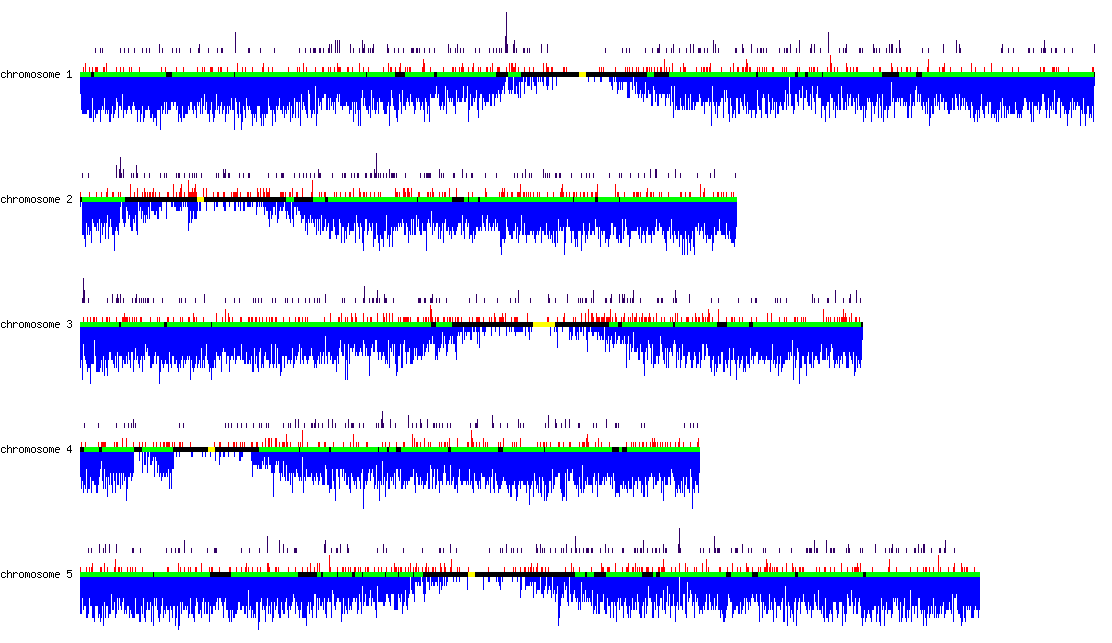

Supplement: Additional file 3 — Distribution of the CBSGs, ALSGs, and ECs within the A. thaliana genome. The five A. thaliana chromosomes are shown with the CBSGs, ALSGs, and ECs plotted in purple, red, and blue from top to bottom, respectively. Segmentally duplicated blocks are indicated in green and the estimated centromeric regions are denoted by a yellow box. [file 1471-2148-10-41-S3.PNG]
